# Corticosteroids and Intravenous Immunoglobulin in Pediatric Myocarditis: A Meta-Analysis

**DOI:** 10.3389/fped.2019.00342

**Published:** 2019-08-16

**Authors:** Yining Li, Yuqing Yu, Selena Chen, Ying Liao, Junbao Du

**Affiliations:** ^1^Department of Pediatrics, Peking University First Hospital, Beijing, China; ^2^Department of Basic Medical Sciences, Peking University School of Basic Medical Sciences, Beijing, China; ^3^Division of Biological Sciences, University of California, San Diego, La Jolla, CA, United States

**Keywords:** myocarditis, children, corticosteroid, intravenous immunoglobulin, meta-analysis

## Abstract

**Background:** The efficacy of corticosteroids and intravenous immunoglobulin (IVIG) in pediatric myocarditis remains controversial.

**Objectives:** The authors performed a meta-analysis to assess the therapeutic efficacy of corticosteroids and IVIG in children with myocarditis.

**Methods:** We retrieved the trials on corticosteroids and IVIG therapy, respectively, in pediatric myocarditis from nine databases up to December 2018. Statistical analysis was performed using Review Manager 5.3.

**Results:** Our analysis included 8 studies and 334 pediatric patients. The data demonstrated that children receiving corticosteroids showed no significant improvement on left ventricular ejection fraction (LVEF) from 1 to 8 month-follow-up (MD = 5.17%, 95% CI = −0.26% to 10.60%, *P* = 0.06), and no significant improvement in death or heart transplantation incidence at the end of follow-up (OR = 1.33, 95% CI = 0.27–6.70, *P* = 0.73). However, children receiving IVIG revealed a statistically remarkable increase in LVEF at a follow-up over the course of 6 months to 1 year (MD = 18.91%, 95% CI = 11.74–26.08%, *P* < 0.00001), and a decrease in death or heart transplantation at the end of follow-up (OR = 0.31, 95% CI = 0.12–0.75, *P* = 0.01). Further comparisons showed that the mortality and heart transplantation rate of children with myocarditis treated with IVIG were significantly lower than those with corticosteroid therapy (*t*' = 11.336, *P* < 0.001).

**Conclusions:** IVIG might be beneficial to improve LVEF and survival for myocarditis in children. However, the present evidence does not support corticosteroids as superior to conventional therapy in children with myocarditis. Further randomized controlled trials with a larger sample size are required.

## Introduction

Myocarditis is generally defined as the inflammatory cellular infiltration in the myocardium and subsequent cardiomyocyte necrosis of non-ischemic origin according to Dallas criteria (1, 2). It has a variety of clinical presentations (3). The exact incidence of myocarditis in children remains unknown (1). A multi-institutional analysis by Klugman et al. showed that myocarditis took up about 0.05% among pediatric hospital discharges in the United States (4). Towbin and colleagues reported that the incidence of dilated cardiomyopathy (DCM) in children was 0.57 cases per 100,000 per year overall, 46% of which were caused by myocarditis (5). It is estimated that the prevalence of pediatric myocarditis is 0.3 cases per year per 100,000 children (6). Although myocarditis is not common in children, it can lead to significant morbidity and mortality (5).

It is generally believed that viral infection is a major cause of myocarditis. The pathophysiology of myocarditis consists of direct viral injury from viral proteins, the innate immune response including cytokines, toll-like receptors, and complements, and the acquired immune response involving T cells and antibodies. A chronic immune reaction related to molecular mimicry may lead to chronic dilated cardiomyopathy even without solid evidence of viral persistence (3, 7–9). Immunosuppressive therapy such as corticosteroids has been used in patients. In experimental models and some uncontrolled cases of myocarditis, intravenous immunoglobulin (IVIG) has shown to have antiviral and immunomodulatory effects (1). However, the efficacy of corticosteroids and IVIG remains controversial. Limited trials have been conducted on children, and the results are inconsistent. We therefore performed a meta-analysis of all qualified randomized or non-randomized controlled trials to determine the therapeutic efficacy of corticosteroids and IVIG in children with myocarditis.

## Methods

This meta-analysis was performed following the Preferred Reporting Items for Systematic Reviews and Meta-Analyses (PRISMA) statement (10).

### Protocol of Searching

Studies were confirmed by searches from medical libraries or electronic reviews of published medical literature in our meta-analysis. The databases, including Cochrane (1943–2018), PubMed (1968–2018), Web of science (1970–2018), EMBASE (1991–2018), Chinese Biomed Database (1978–2018), the Latin American and Caribbean Health Science Information Database (LILACs) (1982–2018), the Cumulative Index to Nursing and Allied Health Literature (CINAHL) (1982–2018), WANFANG (1980–2018), and CNKI (1990–2018), were independently searched by two authors using medical subject heading (MeSH) terms (in English or Chinese) such as “myocarditis/inflammatory cardiomyopathy/dilated cardiomyopathy/carditis AND immunoglobulins/gammaglobulin/gamma-globulin/IVIG,” “myocarditis/inflammatory cardiomyopathy/dilated cardiomyopathy/carditis AND anti-inflammatory agents/glucocorticoids/immunosuppressive agents/adrenal cortex hormones/predniso/dexamethason/hydrocortiso/methylprednison/steroid/corticostero/immunosuppress/ glucocorticoid/mineralocorticoid/betamethason/budesonide/cortiso/fludrocortiso.” We also narrowed the spectrum using the filters of clinical trial and child in the search strategy. No language restrictions were applied. Original articles were acquired from electronic databases or libraries.

### Study Inclusion Criteria

We included English or Chinese studies that referred to randomized or retrospective conventional therapy-controlled trials for the treatment of myocarditis or DCM into our analysis. The diagnosis of myocarditis was made by established clinical, echocardiographic, cardiac MRI, histological, immunological, and immunohistochemical criteria. Trials involving Kawasaki disease, structural heart disease and other specific causes of acute cardiomyopathy as sepsis or drug toxicity were excluded. IVIG, corticosteroids or IVIG in combination with steroid agents were given to the treatment group while traditional therapy was used on the control group. The general characteristics of the subjects, the protocol of the trials and the process of the follow-up were illuminated in detail. Indicators for evaluating effects included the recovery of heart function after undergoing treatment, or the rate of death or heart transplantation during follow-up.

### Study Exclusion Criteria

Studies without a clear standard of diagnosis of myocarditis or the use of other drugs in the control group were not selected. Additionally, studies in which subjects were not children, or the endpoints were not clearly described, were also eliminated. Some uncontrolled trials were excluded as well.

### Study Quality Assessment

Two reviewers independently screened the title and abstract, selected the studies and assessed the quality of studies. Characteristics of subjects, design of trials, course of arrangement and analysis were abstracted and rated. Quality assessment of randomized controlled trials (RCTs) were based on the 7-point Modified Jadad Score, including 7 items on randomization, blinding, allocation concealment, withdrawals and dropouts. Studies were of high quality if they got 4 or more points. The quality of enrolled retrospective cohort studies was evaluated by the 9-star Newcastle-Ottawa Quality Assessment Scale, including 8 items on patient selection, comparability and outcome. Studies were interpreted as high-quality studies if they got 5 or more stars. Divergence was resolved by discussion, or by recourse to a third reviewer. Publication bias was assessed by Funnel plot.

### Statistical Analysis

We used the Review Manager 5.3 software (The Nordic Cochrane Centre of The Cochrane Collaboration, Copenhagen, Denmark) available on international evidence-based medicine cooperative network for meta-analysis. We tested the heterogeneity of study data by forest plot, the *Q*-test as well as *I*^2^ statistics. In the *Q*-test, the results of included studies were homogenous if *p* > 0.05, and a fixed effect model was applied; while if *p* < 0.05, the results were heterogeneous, and the outcome of systematic analysis was stated in a random effects model. *I*^2^ > 50% indicated a significant heterogeneity. The final results were indicated in the form of vector images which combined mean difference (MD) or odds ratio (OR) with 95% confidence interval (CI). We also compared the two medicines by weighted independent *t*-test. *P* < 0.05 showed a statistically significant difference.

## Results

### Basic Data of Included Studies

**Figure 1:**
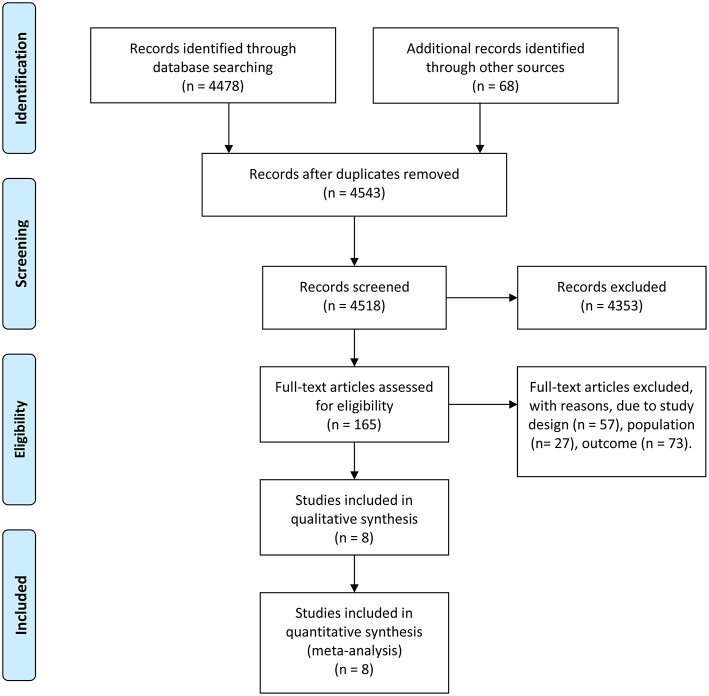
Study flow diagram.

A total of 4,546 studies were retrieved using the retrieval methods mentioned above. Finally, there were eight studies (11–18) being adopted based on exclusion and inclusion criteria (Figure 1), consisting of two randomized controlled trials and six retrospective studies. All studies were published in English. Table 1 shows the general background of eight studies and the list of characteristics of the subjects such as age. In total, 334 pediatric patients with myocarditis or DCM were included, consisting of 146 patients in the experimental group and 188 patients in the control group. The range of mean age of the research subjects was 0–17.7 years. All pediatric patients in these studies were diagnosed as myocarditis or DCM with or without corticosteroids or IVIG. Two of the eight studies regarded only left ventricular ejection fraction (LVEF) as the endpoint of follow-up, while four of the studies used only death or heart transplantation to indicate pharmaceutical effect. The remaining studies used both LVEF and death or heart transplantation to reflect the therapeutic efficacy. In studies mentioning the administration time, the mean time for corticosteroids administration varied from 1 to 8 months, while the range of mean time for IVIG administration was from 2 to 5 days. Follow-up duration lasted from 1 month to 5 years. The results of quality assessment showed that six of the eight studies were high-quality studies (Table 1).

**Table 1:** Basic data of eight included trials.

**References**	**Study design**	**Groups**		**Age**		**Drugs**	**Dosing regimen**	**Control**	**Endpoint**	**Follow-up duration**		***P*-value**	**Quality assessment**
		**T**	**C**	**T**	**C**					**T**	**C**		
Aziz et al. (11)	RCT	44	24	3.4 ± 2.5 years	4.2 ± 3.4 years	Prednisolone	2 mg/kg/day for 1 month and then tapered off over a period of 15 days	Conventional therapy	LVEF	15.1 ± 9.2 months	13.6 ± 10.6 months	0.06	2
Camargo et al. (12)	RCT	12	9	0–15 years		Prednisolone	2.5 mg/kg/day for 1 week, 2.0 mg/kg/day for 3 weeks, 1.5 mg/kg/day for 4 weeks, and 1.0 mg/kg/day thereafter	Conventional therapy	LVEF, death or heart transplantation	8 months		>0.05	3
English et al. (13)	Retrospective	16	6	0–17.7 years		Steroids	2–10 mg/kg/day for a minimum of 3 days	Conventional therapy	Death or heart transplantation	60 months		—	7
Alrabte and Bezanti (14)	Retrospective	13	27	9 months		IVIG	0.4 g/kg/day for 5 days	Conventional therapy	LVEF	12 months		—	7
Atiq et al. (15)	Retrospective	16	20	2.39 ± 3.46 years	2.36 ± 1.75 years	IVIG	A single dose of 2 g/kg	Conventional therapy	Death or heart transplantation	12 months		0.2	9
Haque et al. (16)	Retrospective	12	13	7.3 ± 5.8 years	12.0 ± 4.9 years	IVIG	2 g/kg over 16–24 h on day of admission	Conventional therapy	Death or heart transplantation	—		0.04	7
Heidendael et al. (17)	Retrospective	21	73	10 (1, 51) months^*^	18 (2, 59) months^*^	IVIG	2 g/kg within 2 weeks after initial presentation	Conventional therapy	Death or heart transplantation	33 months		0.432	8
Prasad and Chaudhary, (18)	Retrospective	12	16	<12 years		IVIG	1 g/kg/day for 2 days	Conventional therapy	LVEF, death or heart transplantation	6 months		<0.05	8

* *Age was given as median (interquartile range). Retrospective cohort studies were evaluated using 9-star Newcastle-Ottawa Quality Assessment Scale; RCT studies were evaluated using 7-point Modified Jadad Score; conventional therapy includes digitalis, diuretics, vasodilators, etc. T, treatment; C, control; RCT, randomized controlled trial; IVIG, intravenous immunoglobulin; LVEF, left ventricular ejection fraction*.

### Publication Bias

**Figure 2:**
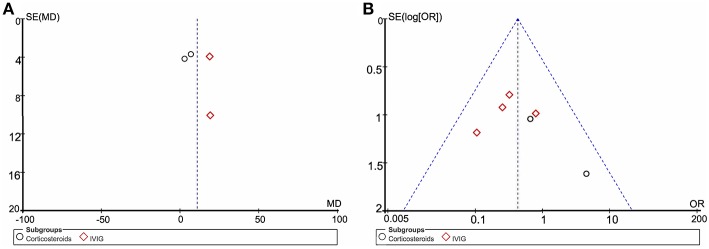
Funnel plot of included studies. **(A)** The left funnel comprised of four dots representing studies using LVEF as the endpoint. **(B)** Funnel plot of six clinical trials using death or heart transplantation as the endpoint. MD, mean difference; OR, odds ratio; SE, standard error.

Funnel plot analysis indicates significant publication bias for the increase in LVEF and death or heart transplantation incidence (Figure 2). The number of articles available is likely a major contributing factor, which also limits our further test for funnel plot asymmetry.

### Analysis of Left Ventricular Ejection Fraction

**Figure 3:**
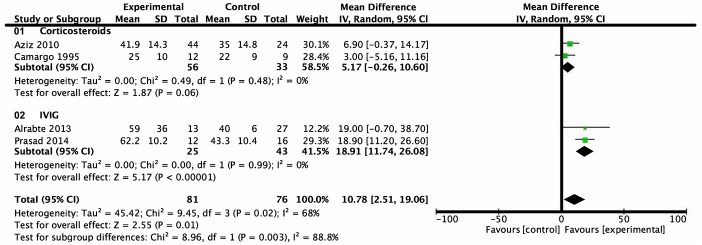
Forest plot of four studies using LVEF as the endpoint. Comparison of drugs and conventional therapy on the outcome of left ventricular ejection fraction in pediatric myocarditis, excluding nonevent trials. Heterogeneity indicated a significant difference (*P* = 0.02, *I*^2^ = 68%). A random effects model was used. CI, confidence interval; SD, standard deviation.

LVEF of children with myocarditis or dilated cardiomyopathy after intervention was reported in four trials. A total of 157 subjects were included, 81 in the treatment group and 76 in the control group. Heterogeneity analysis showed heterogeneity among the studies (*P* = 0.02, *I*^2^ = 68%) and a random effects model was used. Subgroup analysis showed that there was no statistical difference between the group treated with corticosteroids and the group treated with conventional therapy from 1 month- to 8 month-follow-up (mean difference = 5.17%, 95% CI = −0.26%–10.60%, *P* = 0.06). Patients treated with IVIG, on the other hand, revealed a higher level of LVEF compared with patients who received conventional therapy from a follow-up over the course of 6 months to 1 year (mean difference = 18.91%, 95% CI = 11.74–26.08%, *P* < 0.001) (Figure 3).

### Analysis of Death and Transplantation

**Figure 4:**
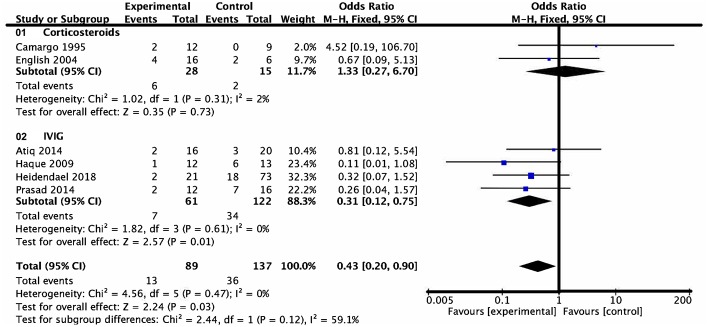
Forest plot of six studies using death or heart transplantation as the endpoint. Drugs vs. conventional therapy on the outcome of rate of death or heart transplantation in pediatric myocarditis, excluding nonevent trials. Heterogeneity showed no significant differences (*P* = 0.47, *I*^2^ = 0%). Fixed effect model for combined effect size was used.

Six studies assigned death or heart transplantation as the endpoint of investigation, in which investigation of 49 patients ended with death or heart transplantation. This consisted of 13 patients in the medication group and 36 patients in the control group. Heterogeneity analysis showed no heterogeneity among the studies (*P* = 0.47, *I*^2^ = 0) and a fixed effect model was used. The death or heart transplantation incidence was lower in the medication group than that in the control group (OR = 0.43, 95% CI = 0.20–0.90, *P* = 0.03). According to the subgroup analysis of the two types of drugs, the death or heart transplantation incidence in pediatric patients treated with IVIG was lower than that of patients treated with conventional therapy at the end of follow-up (OR = 0.31, 95% CI = 0.12–0.75, *P* = 0.01). On the contrary, there was no evidence of a significant difference in the death or heart transplantation incidence between the corticosteroids group and conventional therapy group at the end of follow-up (OR = 1.33, 95% CI = 0.27–6.70, *P* = 0.73) (Figure 4).

Further comparisons showed that the mortality and heart transplantation rate of children with myocarditis treated with IVIG were significantly lower than those with corticosteroid therapy (*t'* = 11.336, *P* < 0.001) (Table 2).

**Table 2:** Comparisons of percentages of death and heart transplantation in children with myocarditis treated by corticosteroids and intravenous immunoglobulin.

**Drugs**	**Reference**	**Percentage of death or heart transplantation (%)**	**Weighted mean percentage (%)**
Corticosteroids	Camargo et al. (12)	16.7	21.4 ± 4.2
	English et al. (13)	25.0	
IVIG	Atiq et al. (15)	12.5	11.5 ± 3.0
	Haque et al. (16)	8.3	
	Heidendael et al. (17)	9.5	
	Prasad and Chaudhary, (18)	16.7	
Weighted independent *t*'-test^*^	*t*' = 11.336, *p* < 0.001		

* *Weighted by number of the subjects; IVIG, intravenous immunoglobulin*.

## Discussion

The results of our meta-analysis show that there is no significant difference in increased LVEF (from a 1 month- to 8 month- follow-up) and decreased death or heart transplantation incidence (at the end of follow-up) between the use of corticosteroids and that of conventional therapy in children with myocarditis or dilated cardiomyopathy. Compared to conventional therapy, additional IVIG treatment may be beneficial for the improvement of LVEF (at follow-up over the course from 6 months to 1 year) and for a decrease in death or heart transplantation rate (at the end of follow-up). The comparison between corticosteroids and IVIG in death or heart transplantation incidence reveals a statistical superiority of IVIG therapy.

Potential etiologies of myocarditis include infections, physical agents, toxins, medications, autoantigens and so on (19). Viral and post-viral myocarditis are major causes of dilated cardiomyopathy (1). The spectrum of viruses varies from enteroviruses (especially coxsackievirus), human herpesvirus 6, adenovirus, Epstein-Barr virus to parvovirus B19, hepatitis C and cytomegalovirus both in children and adults. *Trypanosoma cruzi* is a common cause of myocarditis in Central and South America (20). Our meta-analysis did not include this type of myocarditis because of its distinctive epidemiology, pathogenesis, treatment and management (21). After the initial infection period, patients may develop eosinophilic, lymphocytic, or giant cell/granulomatous inflammation of myocardium (22). Lymphocytic myocarditis is the most common viral myocarditis (9). Manifestations of myocarditis differ in children. Signs and symptoms can be sub-clinical, while patients sometimes experience chest pains similar to pericarditis or myocardial infarction, or even undergo sudden cardiac death from ventricular fibrillation. Moreover, symptoms of heart failure might occur when the disease develops into DCM, leading to death or heart transplantation.

The exact mechanisms for the injury during viral myocarditis have been studied for decades. Research on rodent models and isolated cell systems have shown three phases in the pathophysiology. During phase 1, viruses enter myocytes and macrophages through specific receptors and coreceptors and activate the innate immune response. Phase 2 involves viral replication and an acquired immune response. Protein products of viral genomes can also cause damage to myocardium. Phase 3 is either recovery or DCM. Cellular necrosis triggers the host's immune system and causes further degradation. Molecular mimicry possibly plays an important role in this autoimmune response. On most occasions, the status improves as viral titers decrease, whereas in some cases the disease evolves to chronic dilated cardiomyopathy and become irreversible. It is not clear whether viral persistence or reactivation of latent virus is involved in the chronic phase and eventual onset of DCM. Some viruses, such as parvovirus B19, can also cause myocarditis indirectly by infecting cardiac endothelial cells (1, 3, 9, 23). Genetic predisposition possibly works in viral and/or autoimmune myocarditis and later in DCM in humans (2).

The outcome, prognosis, and efficacy of treatment of myocarditis are closely related to etiology, clinical manifestations and the phase of disease. Conventional therapy includes optimal management of arrhythmia and of heart failure. In patients whose conditions deteriorate, mechanical circulatory support is required, such as extracorporeal membrane oxygenation (ECMO) or ventricular assist devices, as a bridge to recovery or heart transplantation.

As immunosuppressants, corticosteroids could be effective in the second phase of myocarditis with the three-phase pathological course. However, our meta-analysis shows that there is no significant difference in LVEF and death or heart transplantation ratio in children between the corticosteroids group and the conventional treatment group. Corticosteroids use should depend on a prompt diagnosis and a clear assessment of the stage of myocarditis. In the three studies included, however, corticosteroids use varied among different stages of the patients, which likely resulted in no significant differences. In addition, using corticosteroids in acute myocarditis may exacerbate the situation through immunosuppression during the acute viremic phase (11–13). Although several previous randomized controlled trials (24, 25) and meta-analyses (26) have proven the efficacy of corticosteroids, these findings were based primarily on adults. The conclusion may not be conveniently extrapolated to the pediatric population as a result of different etiologies and different physical conditions.

On the other hand, our findings reveal that IVIG may have a therapeutic effect on pediatric myocarditis. Studies have shown that immunoglobulin G and polyvalent intravenous immunoglobulins IgG, IgA, and IgM exert proinflammatory effects, including the activation of immune cells, the complement system, and the opsonization of infective agents. They also have anti-inflammatory effects which comprise the neutralization of bacterial and other toxins, degradation products and an excess of complement factors and cytokines, which help to balance the proinflammatory process (27–29). IVIG can modulate the inflammatory and immune response without major side effects. Thus, if ongoing infection, a post-infectious inflammatory reaction, or a non-infectious process play a role, IVIG can be efficacious (30). However, additional and larger-scale randomized controlled trials on children are necessary for further investigation of IVIG use.

Theoretically, immunosuppressive therapy could lead to side-effects, including infectious diseases, hypertension, edema, an increase in body weight and so on (26). While IVIG therapy was principally associated with infusion-related side effects, all incidences were reported to be mild (30). We also took adverse drug reaction into account, although it was not comprehensively mentioned in the retrieved articles and reflects a limitation of these studies.

Considering the validity of medication targeting different periods of pathologic process, combination therapy may be a more effective option. However, far less research (13, 31) regarding steroid agents combined with IVIG for treating pediatric myocarditis was retrieved. Several studies indicated that combination treatment groups conferred advantages over the control group, while others showed no significant difference in therapeutic effects between the two groups. Therefore, performing more trials to study the efficacy and safety of combined treatment is necessary.

Most previously reported meta-analyses about the treatment for myocarditis (26, 32) targeted adults as research subjects. Moreover, most of these mentioned only IVIG or steroid agents, rather than both. On account of the significant morbidity and mortality rates of pediatric myocarditis, it is of great importance to further investigate more effective therapies. Additionally, our meta-analysis compared the efficacy of corticosteroids and IVIG, which might be taken as a reference for further researches.

## Study Limitations

There were some limitations to the present study. The inverted funnel plot demonstrated an existence of publication bias. It is difficult to unify the diagnostic criteria and most studies lacked a clear assessment of the stage of myocarditis. Several different standards for judging the efficacy of medication were not all-inclusive in involved studies so that we could not estimate it completely. In addition, in biopsy-proven virus-negative patients whose condition deteriorates despite optimal conventional management, immunosuppressive therapy should be considered after ruling out the possible contraindications.

## Conclusions

IVIG might be beneficial to improve LVEF and survival for myocarditis in children. However, the present evidence does not support corticosteroids as superior to conventional therapy in children with myocarditis. Further randomized controlled trials with a larger sample size are required to unambiguously delineate the clinical effect of corticosteroids and IVIG in the treatment of myocarditis in children.

## Data Availability

The datasets generated for this study are available on request to the corresponding author.

## Author Contributions

YLi, YY, and JD contributed conception and design of the study. YLi and YY screened the title and abstract, selected the studies, assessed the quality of evidence, extracted the data, and performed the analysis. SC and YLia supervised study selection and data analysis. YLi and YY drafted the initial manuscript. All authors contributed to manuscript revision and read and approved the submitted version.

### Conflict of Interest Statement

The authors declare that the research was conducted in the absence of any commercial or financial relationships that could be construed as a potential conflict of interest.

## Abbreviations

IVIG: intravenous immunoglobulin
DCM: dilated cardiomyopathy
LVEF: left ventricular ejection fraction
RCT: randomized controlled trial
ECMO: extracorporeal membrane oxygenation
MD: mean difference
CI: confidence interval
OR: odds ratio
SD: standard deviation
SE: standard error.
